# Diabetic Muscle Infarction: An Uncommon Diabetic Complication With a Lack of Standardized Treatment

**DOI:** 10.1210/jcemcr/luad018

**Published:** 2023-02-21

**Authors:** Carlos S Botero Suarez, Moises Matos, Sowmya Suryanarayanan

**Affiliations:** Endocrinology, Diabetes, and Metabolism Fellowship, University of Central Florida HCA Healthcare GME, Greater Orlando, Kissimmee, FL 34741, USA; Department of Internal Medicine, University of Central Florida College of Medicine, Orlando, FL 32827, USA; Endocrinology, Diabetes, and Metabolism Fellowship, University of Central Florida HCA Healthcare GME, Greater Orlando, Kissimmee, FL 34741, USA; Department of Internal Medicine, University of Central Florida College of Medicine, Orlando, FL 32827, USA; Section of Endocrinology, Orlando VA Medical Center, Orlando, FL 32827, USA

**Keywords:** diabetic myonecrosis, diabetic muscle infarction, ESRD, African American, diabetes complication

## Abstract

Diabetic muscle infarction (DMI) is a rare yet serious complication that has been strongly associated with uncontrolled diabetes, although other risk factors are unclear. DMI is an uncommon complication of diabetes with a lack of structured guidelines for evaluation or management. End-stage renal disease (ESRD) could have further implications in patients with DMI in terms of management given that nonsteroidal anti-inflammatory drugs (NSAIDs), which have been shown to reduce the recovery times and recurrence of DMI, could be contraindicated. We present a rare case of DMI in an African American man with ESRD who presented for new-onset right lower-extremity pain and swelling. We discuss the challenges involved with the diagnosis and treatment of this rare condition. This case adds to the knowledge of DMI, which is limited because of the low incidence of this condition, and it helps us understand how this condition affects the African American population and patients with ESRD.

Diabetic myonecrosis, also known as diabetic muscle infarction (DMI), is a rare complication associated with diabetes mellitus. Despite diabetes being a common disease, with a prevalence of more than 400 million worldwide as of 2017, diabetic myonecrosis is a relatively uncommon complication [1]. In a systematic review published in 2015, approximately 126 cases have been reported over 45 years since this condition was first described [2]. Though it was first described in 1965, a standardized management guideline for DMI does not currently exist. Additionally, despite the well-known fact that diabetes mellitus disproportionally affects the African American population, DMI has not been clearly described in the African American population and very little is known regarding the management implications that end-stage renal disease (ESRD) can pose to clinicians. We present a rare case of DMI in a 42-year-old African American man with new-onset right lower-extremity pain and swelling. We also review the pathophysiology, diagnostic workup, and treatment of DMI.

## Case Presentation

A 42-year-old African American man was admitted for evaluation of new-onset right lower-extremity swelling. He had a medical history significant for hypertension, type 2 diabetes mellitus complicated by ESRD requiring hemodialysis, retinopathy requiring laser photocoagulation, and progressive bilateral peripheral neuropathy. The patient had a long-standing history of poorly controlled diabetes mellitus and had been on insulin therapy for more than 10 years but had struggled to adhere to an appropriate regimen. At the time of his admission, he had been on semaglutide, and aspart insulin twice a day and had recently been placed on glargine, but reportedly stopped it because of episodes of hypoglycemia.

**Figure 1.**
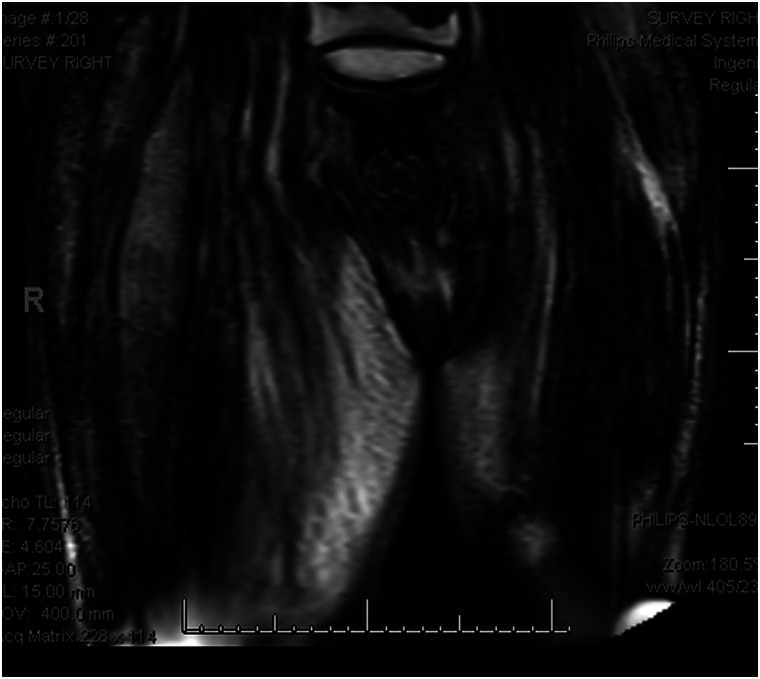
Survey magnetic resonance imaging scan, coronal view of the thighs displaying a size difference of the right and left thigh.

**Figure 2.**
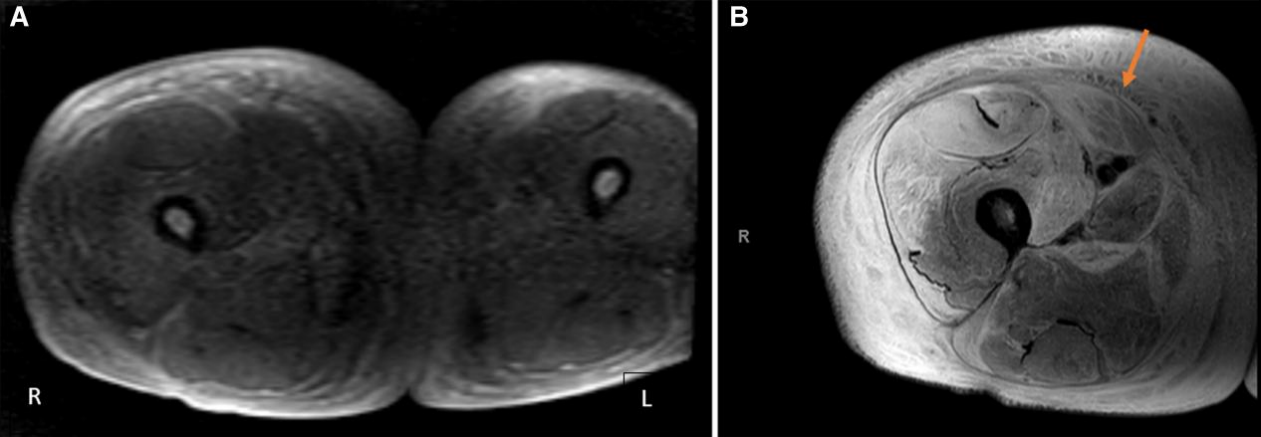
Magnetic resonance imaging survey image showing architectural distortion of anterior fibers of the rectus anterior femoris muscle.

**Figure 3.**
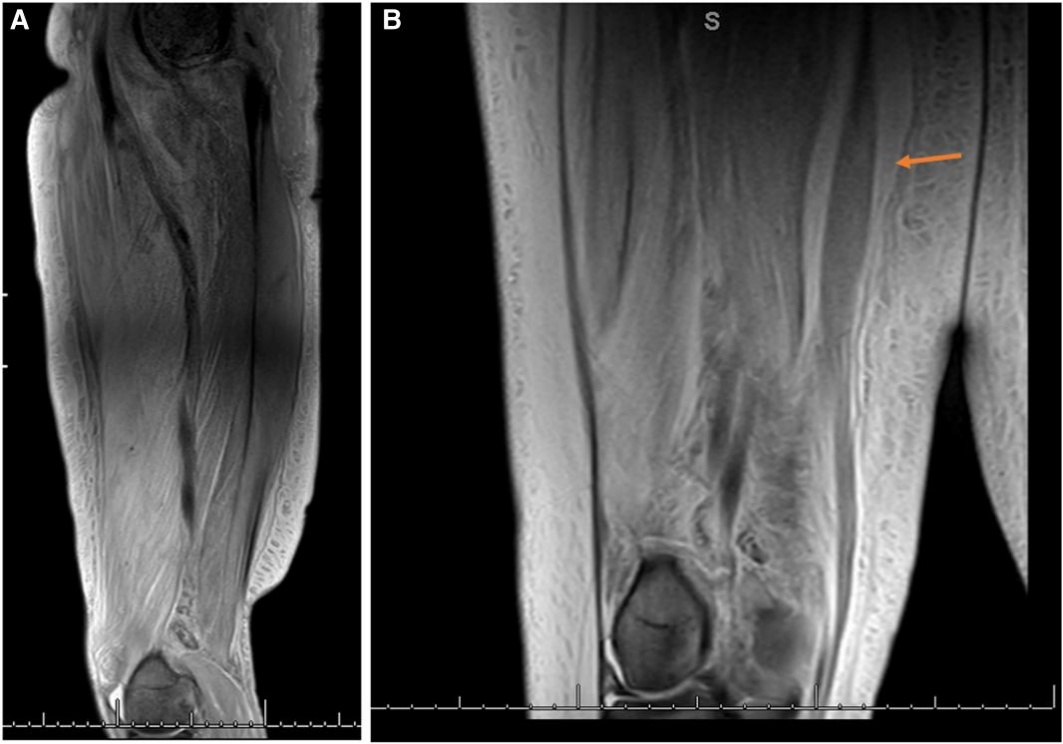
A, Selected coronal T2 magnetic resonance imaging scans showing increased signal of rectus femoris musculature. B, Fluid in the epimysium and along fascial planes (arrow) is most notable around the gracilis muscle indicative of inflammatory changes.

On presentation, the patient reported acute right lower-extremity pain that started 6 days before presentation and was reported as severe, with an intensity of 7 out of 10 using the numerical pain scale. Swelling of the same extremity was also reported and had been gradually worsening for approximately 2 weeks. Vital signs showed a blood pressure of 148/79 mm Hg, heart rate of 81 bpm, respiratory rate of 18 breaths per minute, SPO_2_ 98% on room air, and temperature of 36.6 °C. His physical exam was notable for right lower-extremity induration, tenderness, and swelling of the anterior medial thigh, with pitting edema and warmth to touch along the whole length of the thigh but sparing the inguinal, perineal region, and the knee. Initial laboratory values showed a normal white blood cell count of 9800 cells/mm^3^, slightly elevated D-dimer of 1.52 μ/mL (3040 ng/mL), elevated C- reactive protein of 8.626 mg/dL (821.52 nmol/L), elevated erythrocyte sedimentation rate (64 mm/h), and elevated creatinine kinase (CK) of 381 U/L (6350 nmol/L). His serum creatinine was 6.1 mg/dL (539.24 μmol/L), blood urea nitrogen of 53 mg/dL (18.9 mmol/L), and his electrolytes were within normal limits. At the time of presentation, his glycated hemoglobin A_1c_ was 8.6%. Given his chronic anemia in the setting of ESRD, this value was felt to be inaccurate and thus fructosamine was checked and found to be 517 μmol/L. There were initial concerns for deep vein thrombosis and cellulitis. Therefore, a duplex ultrasound of the right lower extremity was performed, which was negative for deep vein thrombosis. Blood cultures were obtained but remained negative. He completed 6 days of empirical antibiotic therapy with intravenous (IV) vancomycin and clindamycin which was later stopped based on low suspicion of an infectious source. Magnetic resonance imaging (MRI) of the right lower extremity showed marked enlargement of the right thigh (Fig. 1), increased T2 signal compatible with extensive muscle edema in the right thigh, most prominently involving the rectus femoris muscle and adductor musculature with relative sparing of the hamstring muscles (Fig. 2). There was extensive subcutaneous edema, as well as fascial fluid noted (Fig. 3). MRI findings were consistent with diabetic myonecrosis.

## Diagnostic Assessment

Given the patient's history of poorly controlled diabetes, with history, physical examination, laboratory values, as well as MRI findings, the diagnosis of DMI was made on clinical grounds. Biopsy was not performed given the characteristic findings mentioned earlier, as well as previous studies showing lower recovery times when a biopsy is performed [2].

## Treatment

The patient was treated with low-dose aspirin, acetaminophen, and tighter glycemic control.

## Outcome and Follow-up

He was seen in the outpatient clinic 4 weeks after his hospitalization and was started on a continuous glucose monitor. The adoption of the continuous glucose monitor revealed that he had improved glycemic control as noted in his 14-day download record. His glycated hemoglobin A_1c_ (HbA_1c_) improved to 5.8% and his CK levels had decreased to 241 U/L (4016 nmol/L). Further workup revealed negative mitochondrial antibodies (< 20 U) and negative anti-Jo antibodies (< 1.0). The patient was found to have an improvement in the right thigh pain initially, but on further follow-up 3 months after his initial hospitalization, he was found to have recurrence of left thigh pain, swelling, and increased CK levels to 337 U/L (5616 nmol/L). His pain and swelling continued to fluctuate and he could not take traditional analgesics because of ESRD with some residual function. He is awaiting a transplant of his kidneys.

## Discussion

Diabetic myonecrosis, or DMI, was initially reported in 1965 as a rare complication of diabetes mellitus associated with poor control of the disease and microvascular complications [3]. DMI is a rare complication of poorly controlled diabetes mellitus and can occur both in type 1 diabetes mellitus (T1DM) and type 2 diabetes mellitus (T2DM). It is more common in females (54%), occurs with a mean age of presentation of 44.6 years, and has an earlier onset in T1DM when compared to T2DM [2]. More research is needed to determine the predilection for a specific ethnicity. DMI coexistent with ESRD is more common in T2DM (53.7%) than in T1DM (42%). Short-term prognosis of DMI is good, but the recurrence rate is reported to be as high as 43.9% [4]. The mean HbA_1c_ value at the time of diagnosis was 9.34% [2]. Although poor glycemic management is a known risk factor for developing DMI, our patient had an improvement of his HbA_1c_ during his clinic follow-up, yet he later had a recurrence of thigh pain, which may indicate there are other factors at play, such as diabetic microangiopathy, atherosclerosis, and vasculitis [5]. DMI is most often seen in advanced diabetes as demonstrated by studies showing 46.6% of patients had concurrent retinopathy, nephropathy, or neuropathy, and 65% of patients had at least 2 of these complications present [2].

The pathophysiology of DMI remains unclear. Some authors theorize that it is due to diabetic microangiopathy, atherosclerosis, vasculitis with thrombosis, or ischemia-reperfusion injury [6]. DMI should be suspected in patients with poorly controlled diabetes who present with acute muscular pain and swelling. Pain is most common in the thighs, as occurred in our patient, with calf pain and upper arm pain less commonly reported [2]. The most common presentation includes acute, unilateral pain in the quadriceps, local swelling, and an appearance of palpable tender mass, although atypical presentations can be subacute and include bilateral weakness, and motor deficiency with an inability to stand or walk, and requires clinical correlation with imaging and laboratory tests [6]. Trauma is usually not reported, and fever is usually not present [2].

Laboratory evaluation should be guided toward ruling out other differential causes of localized muscle pain, including thrombophlebitis, deep vein thrombosis, trauma, localized myositis, infectious process, abscess formation, or uncommon conditions like tumors, calciphylaxis, and diabetic amyotrophy [6]. Of note, white cell count was noted to be within normal limits in 56% of cases reported, and CK values were reported within normal limits in 68.4% of cases [2]. Workup should also include markers of inflammatory arthritis as this can occur with a similar presentation. Serologic markers such as CK, aldolase, lactate dehydrogenase, aspartate transaminase/ alanine transaminase, anti-Mi-2, anti-Jo1, and anti-SRP can be indicative of inflammatory myositis, including polymyositis, dermatomyositis, and inclusion body myositis. DMI can also elevate inflammatory markers such as C-reactive protein or erythrocyte sedimentation rate, making it challenging to distinguish from inflammatory arthritis [2]. Inflammatory myositis usually presents with proximal muscle weakness and usually pelvic muscles are more severely affected than the shoulder muscles. It usually presents with a chronic onset and little to no associated pain. Characteristically, dermatomyositis presents with additional skin findings including Gottron papules, heliotrope rash, and shawl sign.

Once the suspicion for DMI is high, imaging studies should be performed. Ultrasonography can show heterogeneous, mass-like echogenic changes with loss of normal myofascial interfaces representing muscle swelling, but these findings can be nonspecific. An MRI of the affected extremity is the best imaging study as it can be very specific. Suggestive findings include T1-weighted images showing low-intensity signal enhancement of the affected muscles [7]. There are usually minimal changes in the subcutaneous tissues, which can help distinguish this from cellulitis. T2-weighted images show hyperintensity signals in intramuscular and perimuscular tissues secondary to edema and hemorrhage [8]. Muscle biopsy can be used to confirm the diagnosis but is best reserved for atypical presentations, if other results have been nonconclusive and clinical suspicion remains high, or if treatment fails to elicit improvement [2]. The histologic examination may show signs of muscle necrosis and edematous endomysium, followed by later findings of fibrotic tissue and muscle fiber regeneration, and lymphocytic infiltration. Small-vessel thickening and arteriosclerosis are also evidenced. A biopsy should be avoided when possible given the average time to reported symptoms resolution was significantly longer when a biopsy was performed as opposed to when it was avoided [2]. Therefore, a biopsy is generally discouraged and is reserved for patients in whom a rapidly progressive infection of the muscle cannot be excluded [6]. Additionally, patients with ESRD are at high risk of infection, as this is the second most common cause of mortality in this population. This is due to an impaired innate and adaptive immune system [9]. For this reason, it is reasonable to avoid unnecessary biopsies.

The short-term prognosis of diabetic myonecrosis is good and generally resolves spontaneously over a few weeks. Recovery times from treatment onset are on average 5.5, 8, and 13 weeks using NSAIDs, bed rest with analgesics, and surgical resection, respectively [6]. NSAIDs, including low-dose aspirin, have also been shown to reduce the risk of recurrence of DMI when compared to bed rest alone, physical therapy, or surgery [2]. Tight glycemic control is recommended to reduce the risk of recurrence as this seems to be one of the main risk factors [2]. Recurrence can occur in as often as 35% to 48% of cases, and often involves different muscles group in 39% to 61% of cases [6]. More studies and clinical guidelines are required to guide clinicians in the workup and management of DMI.

## Learning Points

- Diabetic myonecrosis is an uncommon yet important condition that clinicians must recognize given the increasing prevalence of diabetes mellitus.
- Diagnosis of this condition can be challenging because of its broad differential, but a strong clinical suspicion and an MRI with compatible findings can confirm the diagnosis.
- A muscle biopsy can confirm the diagnosis but should be reserved for unclear presentations.
- An accurate clinical diagnosis is important as it results in the avoidance of unnecessary muscle biopsies, antibiotics, or even unnecessary surgeries.
- Diabetic education may need to be broadened to inform patients how to recognize this condition.

## Contributors

All authors made individual contributions to authorship and were involved in the diagnosis and management of this patient. All authors reviewed and approved the final draft.

## Data Availability

Data sharing is not applicable to this article as no data sets were generated or analyzed during the present study.

## Abbreviations

CK: creatinine kinase
DMI: diabetic muscle infarction
ESRD: end-stage renal disease
HbA_1c_: glycated hemoglobin A_1c_
MRI: magnetic resonance imaging
NSAIDS: nonsteroidal anti-inflammatory drugs
T1DM: type 1 diabetes mellitus
T2DM: type 2 diabetes mellitus
